# Transcriptome analysis reveals the proline metabolic pathway and its potential regulation TF-hub genes in salt-stressed potato

**DOI:** 10.3389/fpls.2022.1030138

**Published:** 2022-10-17

**Authors:** Quankai Jing, Hualan Hou, Xiaoke Meng, Airu Chen, Lixia Wang, Husen Zhu, Shuang Zheng, Zhaoyan Lv, Xiaobiao Zhu

**Affiliations:** College of Horticulture, Anhui Agricultural University, Hefei, China

**Keywords:** potato, salt stress, proline, RNA-seq, WGCNA, transcription factor (TF)

## Abstract

Potato (*Solanum tuberosum*) is currently the third most important food crop in the world. However, the production of potato is seriously threatened by salt stress, which often occurs in the facility cultivation environment, and the mining of salt tolerance genes in potato remains to be further studied. In this study, test-tube plantlets of DM potato were treated with 200-mM NaCl to simulate salt stress, and 15 cDNA libraries were constructed for RNA-seq analysis. A total of 8383 DEGs were identified, of which 3961 DEGs were shared among all the salt treatments, and 264 (7.15%) TF-coding genes were identified from these shared DEGs. KEGG enrichment analysis showed that most DEGs identified from the “arginine and proline metabolism” (ko00330) were enriched in the proline metabolic pathway, and their functions almost covered the whole proline metabolic process. Further analysis showed that expression levels of all the 13 structural DEGs in the pathway were significantly up-regulated and proline accumulation was also significantly increased under salt stress, and 13 TF-hub genes were discovered by WGCNA in the lightcyan and tan modules which were highly positively correlated with the proline contents. Correlation analysis revealed that the four TF-hub genes of the lightcyan module and seven structural DEGs of the proline metabolic pathway might be the potential candidate genes, especially the potential and novel regulatory gene *StGLK014720*. Furthermore, the dual-luciferase reporter assay confirmed that the key protein StGLK014720 could activate the promoters of both structural genes *StAST021010* and *StAST017480*. In conclusion, these results lay the foundation for further study on the salt tolerance mechanism of potato, and provide a theoretical basis and new genetic resources for salt tolerance breeding of potato.

## Introduction

Potato (*Solanum tuberosum*) is the world’s most important non-cereal food crop, belonging to the *Petota* section of the *Solanum* genus within the Solanaceae family (Spooner et al., 2014; Tang et al., 2022). Along with the unceasing increase in the world population, people have put forward higher requirements on crop yields. Potato is cultivated in more than 100 countries and over a billion people worldwide eat it (Dahal et al., 2019), so increasing potato productivity is crucial to global food security. However, potato production is often restricted by various abiotic stresses, among which salt stress is one of the major constraints. Soil salinization is a worldwide problem that threatens the growth and yield of crops and hinders the sustainable development of modern agriculture (Zhao et al., 2020). Crop yield loss due to soil salinization is a growing threat to agriculture worldwide.

Salinity stress imposes osmotic stress and ion toxicity, which impairs major plant processes such as photosynthesis, cellular metabolism, and plant nutrition (Joshi et al., 2022). Osmotic stress triggers signaling pathways that promote the biosynthesis and accumulation of compatible osmolytes, which is important for both short-term and long-term osmotic stress tolerance in plants (Zhao et al., 2020). Some of the osmolytes, such as proline, glycine betaine, polyamine, mannitol, glucose, fructose, and trehalose, are typically accumulated in several plant species in response to salt, heat, drought, ionic, osmotic, and heavy metal stresses (Gul et al., 2022). Recent evidence from salinity tolerance mechanisms highlights that osmolytes play a key role in quenching free radicals, inducing antioxidant machinery, and osmotic regulation (Choudhary et al., 2022). These osmolytes participate in the regulation of osmotic pressure by lowering the osmotic potential in the cytosolic compartment (Zhao et al., 2021). All crop plants contain proline, which increases as the plants are subjected to various stressors (Gul et al., 2022).

The increase of proline content in plants is usually involved in response to environmental stress. Proline is not only a signal substance, but can also improve plant resistance to abiotic stress by improving photosynthesis, antioxidant activity, osmotic pressure, and regulating the homeostasis of sodium and potassium (Hosseinifard et al., 2022). The accumulation of proline can be achieved through the activation of the proline biosynthetic pathway and the inactivation of the catabolic pathway (Kiyosue et al., 1996). In potato, PEG simulated drought stress promoted the up-regulated expression of Δ-1-pyrroline-5-carboxylate synthase (*P5CS*) and pyrroline-5-carboxylate reductase (*P5CR*) genes in the proline biosynthesis pathway, and inhibited the expression of pyrroline dehydrogenase (*PDH*) and Δ-1-pyrroline-5-carboxylate dehydrogenase (*P5CDH*) genes in the proline catabolic pathway, and the expression levels of *P5CS* and *P5CR* genes in the proline biosynthesis pathway were positively correlated with the proline contents (Liu et al., 2019). Over-expression of the *AtP5CS* gene could significantly improve the proline contents and salt tolerance of potato transgenic lines (Hmida-Sayari et al., 2005). A previous study has shown that the potato variety Desiree was more salt-tolerant than Mozart, and the expression level of the *P5CS1* gene was significantly up-regulated in Desiree under salt stress, while there was no significant change in Mozart (Jaarsma et al., 2013).

In addition, several genes, which were not involved in the proline metabolic pathway, could also enhance plant salt tolerance by promoting proline accumulation. El-Banna et al. (2010) demonstrated that transgenic suspension cells of the potato cultivar Desiree over-expressing the PR-10a protein could significantly improve the proline levels and salt tolerance compared to the wild-type cells, respectively. Transgenic Arabidopsis lines, which over-express glyceraldehyde 3-phosphate dehydrogenase (GAPDH) encoded by the *StD43* gene, showed a significantly higher proline content and salt tolerance than those in the wild-type (WT) lines (Kappachery et al., 2021). Over-expression of the *StCYS1* gene in transgenic potato plants significantly promoted their tolerance to high salinity and accumulation of proline and chlorophyll under salt stress (Liu M. et al., 2020). A previous study has found that different families of transcription factors (TFs) could activate the expressions of proline biosynthetic genes (Zarattini and Forlani, 2017). Ectopic expression of dehydration-responsive element binding proteins (StDREB2) proved that *StDREB2* might be involved in the potential response process of plants to abiotic stress by regulating the transcription level of gene *P5CS* to promote proline accumulation (Bouaziz et al., 2012).

Plants in response to different stresses are highly complex and involve changes at the transcriptome, cellular, and physiological levels, and involve the synergistic action of multiple genes, but previous studies on potato salt stress mainly focused on the physiological, biochemical, and functional verification of a few genes (Fidalgo et al., 2004; Levy and Veilleux, 2007; Chourasia et al., 2021). Moreover, transcriptome researches mostly focused on the identification of DEGs but rarely on elucidating gene regulatory networks, and transcriptome research for multiple time points (within 24 h) at the initial stress stages has not been reported. In this study, to gain further insights into the response mechanisms of potato to salt stress and mining key candidate genes that can improve potato salt tolerance, the DM test-tube plantlets treated with NaCl were used for RNA-seq analysis, and WGCNA was combined together to construct co-expression networks. Then multiple gene modules in the networks and TF-hub genes in each module were identified, respectively. Finally, the TF-hub genes with potential regulatory effects on the proline accumulation were screened according to the expression patterns of the DEGs in the proline metabolic pathway.

## Materials and methods

### Plant materials and salt treatment

The tissues of the doubled monoploid potato clone DM1-3 516 R44 (DM, a homozygous line, 2*n* = 2*x* = 24) were cultured in test tubes containing 10 mL Murashige and Skoog solid medium (MS, 3% (w/v) sucrose, pH 5.6-5.8) in the light incubator with a 16-h light/8-h dark cycle, day/night temperatures of 22°C/18°C (Hardigan et al., 2016; Zeng et al., 2019).

The concentration of NaCl treatment was the same as that in previous studies (Zhou et al., 2018; Zhu et al., 2021). The transcript levels of the previously reported NaCl-responsive genes *StZFP1* (Tian et al., 2010; Liu Z. et al., 2020), *StMAPK3* (Zhu et al., 2020), and *StDWF4* (Zhou et al., 2018) were used to check the stress intensity of the test-tube plantlets to salt stress. 45-day-old test-tube plantlets were treated with 10 mL of liquid MS medium containing 400-mM NaCl (the treatment group, the final concentration of NaCl was 200 mM), and MS liquid medium as the control. After 24 h of ion balance, the treatment solutions in the test tubes were removed. At 3 h, 6 h, 12 h, and 24 h (S1, S2, S3, and S4) after the ion balance, the aboveground parts of DM test-tube plantlets were quickly cut off and put into liquid nitrogen immediately, then transferred to -80°C for cold storage, and the control group (CK, 10 mL MS) was collected at 24 h after the ion balance. The experiment was repeated three times with three biological replicates and four test-tube plantlets for each replicate.

### RNA extraction and quality assessment

Total RNAs were extracted from the aboveground parts of DM test-tube plantlets from each NaCl treatment, using OmniPlant RNA Kit (DNase I) (CW2598S, CWBIO, China) following the manufacturer’s instructions. RNA concentrations and purities were measured using NanoDrop 2000 (Thermo Fisher Scientific, Wilmington, DE). RNA integrities were assessed using the RNA Nano 6000 Assay Kit of the Agilent Bioanalyzer 2100 system (Agilent Technologies, CA, USA).

### qRT-PCR analysis

Methods of qRT-PCR (quantitative real-time PCR) for gene expression analysis were applied according to our previously published protocols (Zhu et al., 2014; Zhu et al., 2016). The first-strand cDNA was synthesized from RNA used in RNA-seq by using PrimeScript™ RT reagent Kit with gDNA Eraser (RR047A, Takara, Japan), and then qRT-PCR was performed using TB Green^®^ Premix Ex Taq™ I (RR820A, Takara, Japan). 16 genes were used for qRT-PCR assays to validate the reliability and accuracy of the RNA-seq data. The *StActin97* was used as the reference gene for gene expression normalization. The 2^−ΔΔCt^ method was used to calculate the relative expression levels of the genes. Each gene was examined in three biological replicates. The primer sequences used in this study are listed in **Supplementary Table 1**.

### Library establishment and sequencing

A total of 1 μg RNA per sample was used as input material for the RNA sample preparations. Sequencing libraries were constructed by NEB Next UltraTM RNA Library Prep Kit for Illumina (NEB, USA). To select cDNA fragments of preferentially 240 bp in length, the library fragments were purified with the AMPure XP system (Beckman Coulter, Beverly, USA). Library quality was assessed on the Agilent Bioanalyzer 2100 system.

The clustering of the index-coded samples was performed on a cBot Cluster Generation System using TruSeq PE Cluster Kit v4-cBot-HS (Illumia) according to the manufacturer’s instructions. After cluster generation, the libraries were sequenced using the Illumina platform and paired-end reads were generated.

### Quality control of RNA-seq data

Raw data (raw reads) of fastq format were firstly processed through in-house Perl scripts. In this step, clean data (clean reads) were obtained by removing reads containing adapter, reads containing ploy-N and low-quality reads from raw data. At the same time, Q20, Q30, GC-content, and sequence duplication levels of the clean data were calculated. All the downstream analyses were based on clean data with high quality.

The principal component analysis (PCA) was performed using the PCAtools package in the R software (Kyritsis et al., 2021).

### Alignment and annotations of new transcripts

The new transcripts were found by filtering out the sequences of short peptide chains (less than 50 amino acid residues) or single exons. The DIAMOND software was used to compare the new genes with NR, Swiss prot, COG, KOG, and KEGG databases, and then the new genes were analyzed with the GO database by InterPro (Jones et al., 2014; Buchfink et al., 2015). After predicting the new gene, HMMER software was used to compare it with the Pfam database (Eddy, 1998; Punta et al., 2011).

### Quantitative gene expression, differentially expressed gene analysis, and prediction of TFs and TFBs

Raw sequences were transformed into clean reads after data processing, then these clean reads were mapped to the reference genome (DM v6.1) using the Hisat2 software. Gene expression levels were estimated by fragments per kilobase of transcript per million mapped reads (FPKM). A formula for the calculation of the FPKM was shown as follows:

FPKM=cDNA Fragments/(Mapped Fragments (Millions) * Transcript Length (kb)).

Differential expression analysis of each treatment was performed using the DESeq2 software. Genes with an adjusted *P*-value < 0.01 (FDR < 0.01) and Fold Change ≥ 2 found by DESeq2 were assigned as DEGs.

The information of TF families was predicted using Plant TFDB (http://planttfdb.cbi.pku.edu.cn/). TF binding sites (TFBs) in the promoter region were detected by using the “promoter analysis” function of the PlantPAN 2.0 database (http://plantpan.itps.ncku.edu.tw/promoter.php) (Chow et al., 2015; Zarattini and Forlani, 2017).

### Enrichment analysis

Gene Ontology (GO) enrichment analysis of the differentially expressed genes (DEGs) was implemented by the GOseq R packages based on the Wallenius non-central hyper-geometric distribution (Young et al., 2010), which could adjust for gene length bias in DEGs.

Kyoto Encyclopedia of Genes and Genomes (KEGG) is a database resource for understanding high-level functions and utilities of the biological system, such as the cell, the organism, and the ecosystem, from molecular-level information, especially large-scale molecular datasets generated by genome sequencing and other high-throughput experimental technologies (http://www.genome.jp/kegg/) (Kanehisa et al., 2007). We used KOBAS software to test the statistical enrichment of DEGs in KEGG pathways (Mao et al., 2005).

### Determination of proline and malondialdehyde contents

The contents of proline and malondialdehyde (MDA) in a total of 15 samples (S1, S2, S3, S4, and CK) were measured by Proline Determination Kit (colorimetry) (A107-1-1, Nanjing Jiancheng, China) and Plant Malondialdehyde Determination Kit (microplate method) (A003-3-1, Nanjing Jiancheng, China) following the manufacturer’s protocols, respectively.

### Weighted gene co-expression network analysis

The WGCNA package in R software was used to construct a gene co-expression network (FPKM ≥ 1, and variation of FPKM: cv ≥ 0.5), and the physiological indexes and gene expressions of the DEGs were analyzed in this study. The graphical network was created by Cytoscape 3.7.1 software for each module (weight ≥ 0.1) (Shannon et al., 2003).

Hub genes in each co-expressed module were defined according to module eigengene-based intramodular connectivity or module membership (kME) index in non-preserved modules (Panahi and Hejazi, 2021). Genes with |kME| ≥ 0.7 were considered as hub genes in the respective module (Langfelder and Horvath, 2008). The ggcor package in R software was used for correlation testing and visualization (Yang et al., 2021).

### Dual luciferase reporter assay

A 711-bp full-length cDNA fragment of the potential key regulatory gene *StGLK014720* was cloned and recombined into the pGreen II 62-SK vector to generate an effector. 1,500-bp DNA fragments (upstream of TSS) of promoter regions of the seven DEGs in the proline metabolic pathway were cloned and inserted into the pGreenII 0800-LUC vector to obtain different reporters (Luo et al., 2022). The recombinant plasmids were transformed into the *Agrobacterium Tumefaciens* strain GV3101 (pMP90). The leaves of 4-week-old tobacco plants (*Nicotiana benthamiana*) were subjected to transient expression assays according to the previous methods (Cheng et al., 2022). The activities of Firefly Luciferase (LUC) and Renilla Luciferase (REN) were determined by using the Dual Luciferase Reporter Gene Assay Kit (11402ES60, Yeasen, China) according to the manufacturer’s instructions. The ratio of LUC/REN showed the activity of the promoter. The tobacco leaves co-transformed with the reporter and pGreenII 62-SK vector without *StGLK014720* were used as the control, the LUC/REN ratio of which was taken as 1. Constructs for dual luciferase reporter assay were developed using primers mentioned in **Supplementary Table 2**.

## Results

### Transcriptional responses and transcriptome quality assessment

**Figure 1:**
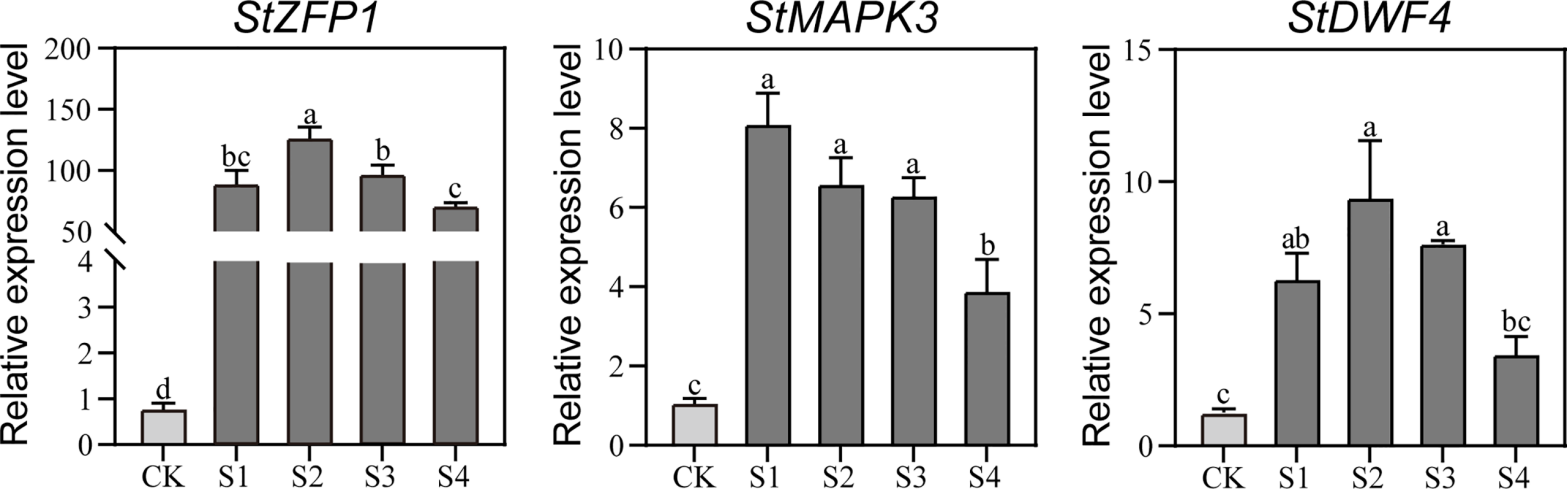
The qRT-PCR-based transcription analysis of the three NaCl-responsive genes in the DM plantlets under NaCl stress. Gene expression is normalized relative to the potato reference gene *StActin97*. Data presented as mean ± standard error from three biological replicates were tested by one-way analysis of variance (ANOVA). Different letters represent statistically significant differences (*α* = 0.05). S1, S2, S3, and S4, represent the aboveground parts of DM text-tube plantlets under 200-mM NaCl treatment for 3, 6, 12, and 24 h, respectively; CK represents the aboveground parts of DM text-tube plantlets after 24 h treatment with MS liquid medium (the same below).

To check the transcriptional responses of the aboveground parts of DM test-tube plantlets under the 200-mM NaCl treatments, three NaCl-responsive genes previously reported as *StZFP1*, *StMAPK3*, and *StDWF4* were selected for qRT-PCR assays. The results showed that the expression levels of the three representative genes were significantly up-regulated, and then significantly decreased at 12 h or 24 h after the NaCl treatments (**Figure 1**), which indicated that the 200-mM NaCl treatments were suitable for inducing salt stress to the plantlets.

To comprehensively understand the transcriptional responses to salt stress in potato, 15 cDNA libraries were constructed for RNA-seq analysis. After cleaning and quality control, a total of 140.29 Gb of sequencing data was obtained by Illumina Hi-seq 2000 platform (**Supplementary Table 3**). The data of all 15 samples were more than 7.31 GB, the Q30 bases accounted for greater than 91.49% of the total, and the GC contents were between 42.97% and 43.60% (**Supplementary Table 3**). The *S. tuberosum* DM v6.1 database was used as the reference genome, and the alignment results showed that 93.34%~96.72% of the reads were mapped to the reference genome (**Supplementary Table 4**). To verify the repeatability and reliability of the results, we performed principal component analysis (PCA) on the gene expression data of 15 samples. The analysis of the expression patterns showed that there were significant separations between NaCl-treated samples and the controls, and between samples at different treatment times, while the samples from biological replicates grouped together (**Supplementary Figure 1**).

To validate the reliability and accuracy of the RNA-seq data, and to confirm the expression patterns of the structural DEGs and their potential regulatory DEGs in the proline metabolic pathway, thirteen DEGs potentially related to proline metabolism and three NaCl-responsive genes were also selected for qRT-PCR assays. The results showed that the qRT-PCR results of the 16 genes were consistent with the RNA-seq data, demonstrating the accuracy of the RNA-seq data (**Supplementary Figure 2**).

### Identification of novel transcripts, DEGs, and TF-encoding genes under salt stress

**Figure 2:**
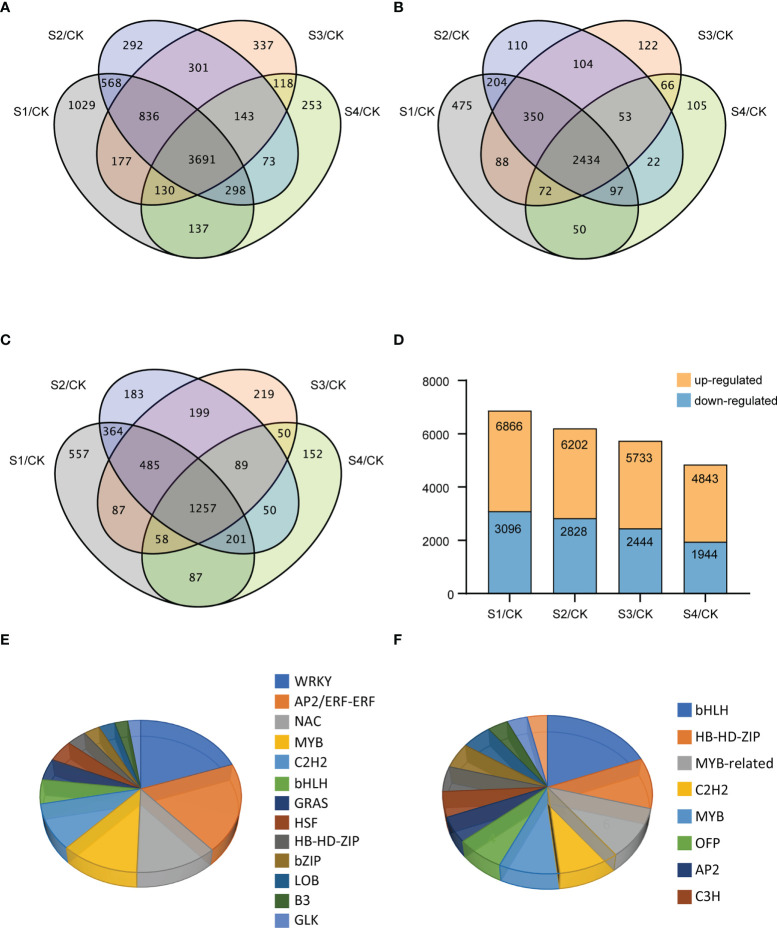
Differentially expressed genes (DEGs) and TF-coding genes after 3, 6, 12, and 24 h of NaCl stress in the DM plantlets. **(A–C)** Venn diagrams for all, up-, and down-regulated genes, respectively. **(D)** Distribution diagram for the numbers of the DEGs at different salt treatment times, and all NaCl-treated samples were compared with CK samples. **(E, F)** Pie charts for the numbers of up-regulated and down-regulated genes in different TF families of potato under salt stress, respectively.

A total of 3716 novel transcripts were identified from the RNA-seq data, and 1920 new genes were functionally annotated (**Supplementary Table 5**). To identify DEGs among different treatments under salt stress, a total of 8383 DEGs were revealed by comparing and analyzing the RNA-seq data of NaCl-treated samples at different treatment times and the control. The Venn diagram indicated the numbers of the unique and shared DEGs under 200-mM NaCl treatments at different times (**Figure 2A**). We cross-compared the DEGs among the four different datasets *vs*. CK, which resulted in 3691 shared DEGs, comprised of 2434 up-regulated and 1257 down-regulated genes. Among the regulated genes under salt stress, 1029, 292, 337, and 253 genes were exclusively expressed at 3 h, 6 h, 12 h, and 24 h, respectively. As shown in **Figure 2B**, 2770, 3374, 3289, and 2899 genes were significantly up-regulated after NaCl treatment for 3 h, 6 h, 12 h, and 24 h, respectively. Among the down-regulated genes, the expressions of 3096, 2828, 2444, and 1944 genes were regulated specifically at 3 h, 6 h, 12 h, and 24 h, respectively (**Figure 2C**). In general, most DEGs showed up-regulated expression in samples treated with NaCl at four different time points (**Figure 2D**).

A total of 264 (7.15%) TF-coding genes were predicted in the 3691 DEGs, of which 198 genes were up-regulated while 66 genes were down-regulated (**Figures 2E, F** **)**. These TFs mainly included AP2/ERF, WRKY, MYB, and NAC families, and the corresponding numbers of family members were 36, 35, 25, and 24, respectively. Further analysis revealed that the WRKY family had the largest number of up-regulated TF-coding genes (34) (**Figure 2E**), while the bHLH family had the largest number of down-regulated TF-coding genes (11) (**Figure 2F**).

### Enrichment analysis of the DEGs

To understand the biological functions and molecular responses in metabolic pathways of potato DEGs under salt stress, enrichment analysis of the DEGs was carried out in this study based on the GO and KEGG databases. Among the shared 3691 DEGs in all differential groups, 2936 and 2500 genes could be annotated by GO and KEGG databases, respectively.

GO enrichment analysis of these annotated DEGs shows that the DEGs participated in three categories: “biological process, BP”, “cell composition, CC” and “molecular function, MF” (**Supplementary Figure 3**). “Cell process” and “metabolic process” were the most obvious terms in the biological process category; “Binding” and “catalytic activity” were the most enriched in the metabolic process category; in the cell composition category, “membrane” had the largest number of genes. Notably, the distribution pattern of DEGs annotated in the GO database was different from all potato genes. A higher proportion of DEGs annotated in the GO database were enriched in “detoxification, BP”, “antioxidant activity, MF”, and “nucleic acid binding transcription factor activity, MF”.

We identified the KEGG pathway with the largest proportion of the DEGs in potato under salt stress (KEGG ontology terms, *P* < 0.05) (Chen et al., 2021). NaCl treatment significantly affected metabolic pathways in the aboveground parts of DM test-tube plantlets, including “photosynthesis-antenna proteins”, “carbon fixation in photosynthetic organizations”, “phenylalanine metabolism”, and “glutathione metabolism” (**Supplementary Figure 4**).

**Figure 3:**
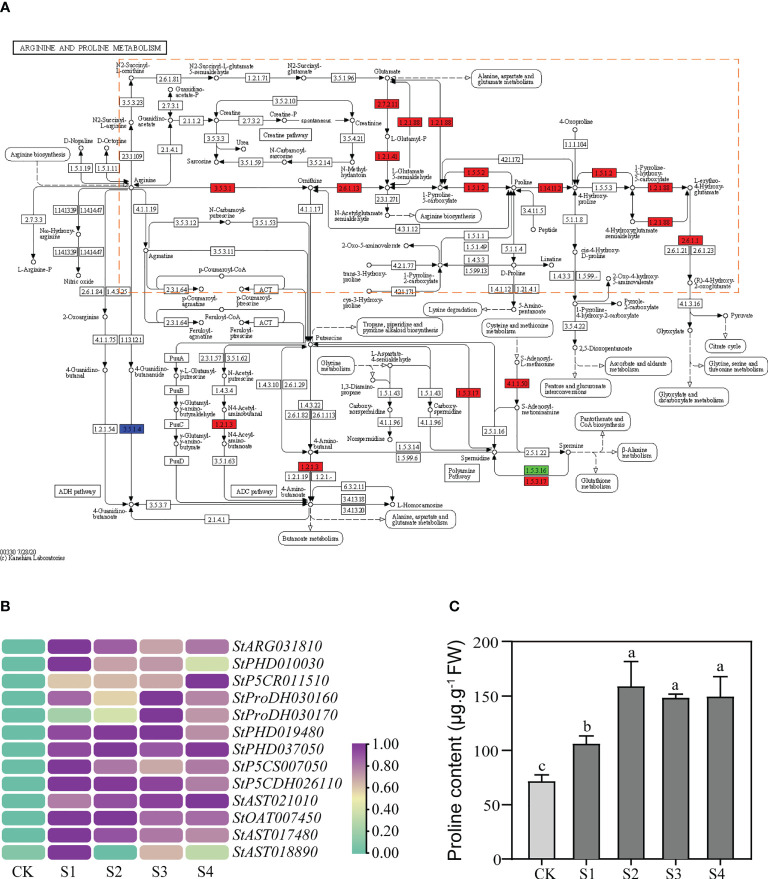
Expression levels of the structural DEGs in the proline metabolic pathway and the proline contents in the DM plantlets under salt stress. **(A)** Differential expression of the structural DEGs in KEGG enrichment analysis. The green rectangle represents the DEGs down-regulated; the red rectangle represents the DEGs up-regulated; the blue rectangle represents both up-regulated and down-regulated DEGs. The proline metabolic pathway is highlighted by orange dotted frame. **(B)** Heat map of the structural DEGs in the proline metabolic pathway. **(C)** Changes of the proline contents in aboveground parts of DM text-tube plantlets under the 200-mM NaCl treatments. Data presented as mean ± standard error from three biological replicates were tested by one-way analysis of variance (ANOVA). Different letters represent statistically significant differences (*α* = 0.05).

KEGG enrichment analysis showed that a total of 19 DEGs were enriched in the “arginine and proline metabolism” (ko00330) pathway, of which up to 13 DEGs were concentrated in the proline metabolic pathway (**Figure 3A**). The 13 DEGs included not only main hot-spot genes in proline research, such as *P5CS*, *P5CR*, *ProDH*, and *P5CDH*, but also many genes that have received little attention, such as *ARG* and *AST* (**Supplementary Table 6**). Overall, we found that many genes in the proline metabolic pathway were up-regulated under salt stress, and their functions almost covered the whole proline metabolic process (**Figure 3A**; **Supplementary Table 6**).

### Salt stress changed the expression levels of the proline metabolic genes and the proline contents

Further results showed that expression levels of all the 13 structural DEGs in the proline metabolic pathway were significantly up-regulated under salt stress (**Figure 3B**). The expression levels of 5 (35.7%) of the 13 structural DEGs peaked at 12 h or 24 h and increased 1.96~7.66 times more than that of the controls. These structural DEGs comprised *StP5CR011510*, *StProDH030160*, *StProDH030170*, *StPHD019480*, and *StAST021010*, while the peaks of the expression levels of the remaining 8 structural DEGs appeared in advance at 3 h or 6 h and the expression levels increased 1.96~7.66 times more than that of the controls (**Figure 3B**).

To verify the correlation between the expression levels of the proline metabolic genes and the proline contents, we measured the proline contents in DM plantlets under salt stress, and the content of free proline can also be used as an indicator to reflect the osmotic adjustment system. Meanwhile, we also measured MDA content as the marker to measure oxidative damage. The proline contents increased gradually after 200-mM NaCl treatments and reached maximum at 6 h, which was 157.78 μg·g^-1^ and 2.22 times higher than the control, then stabilized with extension of time (**Figure 3C**). The results indicated that salt stress significantly promoted proline accumulation in the aboveground parts of DM test-tube plantlets. However, the MDA contents did not change significantly with the extension of time (**Supplementary Figure 5**).

### WGCNA analysis and TF-hub genes identification

**Figure 4:**
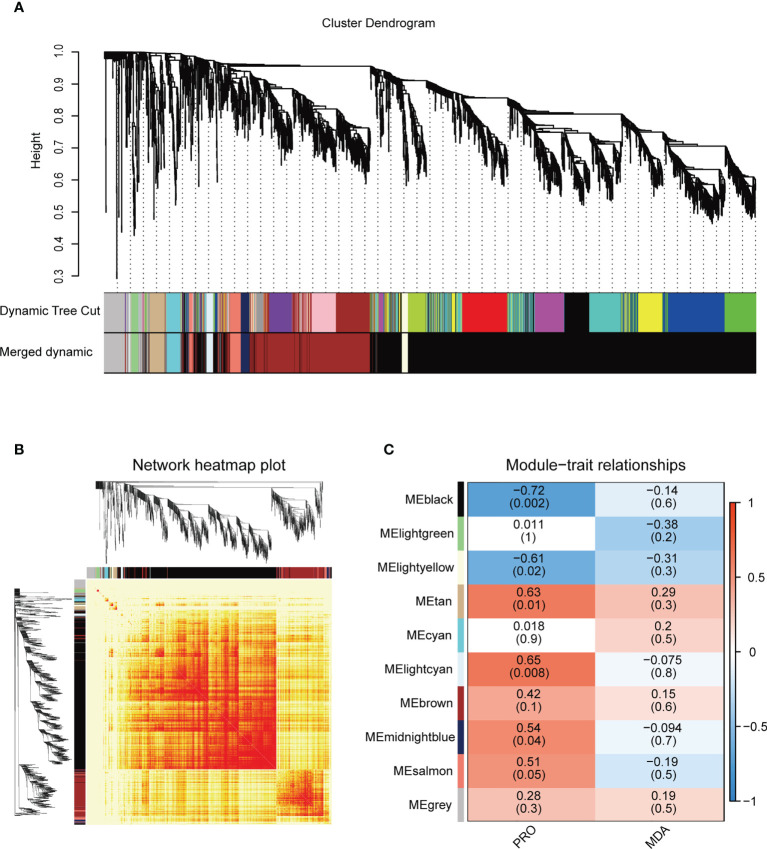
Weighted gene co-expression network analysis (WGCNA) of salt stress-responsive genes in the DM plantlets under salt stress. **(A)** Hierarchical cluster tree of the common genes between different studies. The branches and color bands represent the assigned modules; the tips of the branches represent genes. **(B)** The heat map shows the Topological Overlap Matrix (TOM) value among the proteins of the network delimited into modules by the dynamic method. Lower TOM is indicated by the yellow color and higher TOM is indicated by the progressively red color. **(C)** The thermogram showed the correlations between the modules and the contents of proline and malondialdehyde. No significant correlations are displayed in white, positive correlations and negative correlations are displayed in red and blue, respectively. PRO, proline; MDA, malondialdehyde (the same below).

WGCNA analysis was used to identify the co-expressed gene modules and construct the interrelationship network between genes, and to reveal the hub genes in response to salt stress. A co-expression network was generated according to the expression of the DEGs in 15 samples, the genes with similar expression patterns were assigned into the same module, and finally a total of 10 different modules were identified (**Figures 4A, B**). The spatio-temporal specific modules lightcyan and tan were significantly positively correlated with the proline contents (*R* = 0.63~0.65, *P* = 0.008~0.01) (**Figure 4C**). The genes with |kME| ≥ 0.7 were regarded as the hub genes of the module, and the hub genes represent the overall functionality of the module. Combined with gene domain analysis, the hub genes with TF domains (TF-hub genes) were identified in the modules.

A total of 44 hub genes were delimited in the lightcyan module, which were expressed at low levels in the control samples (CK), but had the highest expression levels in the NaCl-treated samples at 24 h (S4) (**Supplementary Figure 6A**). KEGG enrichment analysis indicated that these genes slowly responded to salt stress (24 h) and were mainly involved in the synthesis pathways of secondary metabolites such as “phenylpropanoid biosynthesis, ko00940” and “flavonoid biosynthesis, ko00941”, the two most significantly enriched metabolic pathways. The lightcyan module contained five TF-hub genes: GLK family encoding gene *StGLK014720*, NAC family encoding gene *StNAC015310*, WRKY family encoding gene *StWRKY026100*, C2H2 family encoding gene *StC2H2001820*, and MADS-MIKC family encoding gene *StMADS036110* (**Supplementary Table 7**). The Cytoscape software (weight ≥ 0.1) was used to create a visual gene interaction network in the lightcyan module (**Supplementary Figure 6B**). Hub genes with more connections in the network might be the key regulatory genes. The TF-hub genes, except for *StC2H2001820*, were located in the visual gene interaction network of the lightcyan module.

A total of 68 hub genes were found in the tan module, and most of them responded to salt stress at 6 h and 12 h (**Supplementary Figure 7A**). GO enrichment analysis showed that the hub genes in the tan module were significantly enriched in “iron ion binding, BF”. KEGG enrichment analysis found that the most significantly enriched pathways were “plant hormone signal transduction” (ko04075), suggesting that the hub genes within the module respond to salt stress perhaps *via* regulating plant hormones. There were eight TF-hub genes in the tan module, including C2C2-CO-like family encoding genes *StCOL012520* and *StCOL023800*, GARP-G2-like (GLK) family encoding genes *StGLK031660* and *StGLK003930*, bHLH family encoding gene *StbHLH026970*, bZIP family encoding gene *StbZIP015000*, HSF family encoding gene *StHSF025050* and B3-ARF family encoding gene *StARF010540* (**Supplementary Table 8**). To further understand the regulatory genes in the tan module, a visual gene interaction network was constructed by using Cytoscape software (weight ≥ 0.1), in which each node represents a gene and the connecting lines (edges) between genes represent co-expression correlations (**Supplementary Figure 7B**). The results showed that all eight TF-hub genes were located in the interaction network of the tan module.

### Screening of the structural DEGs and their potential regulatory TF-hub genes

**Figure 5:**
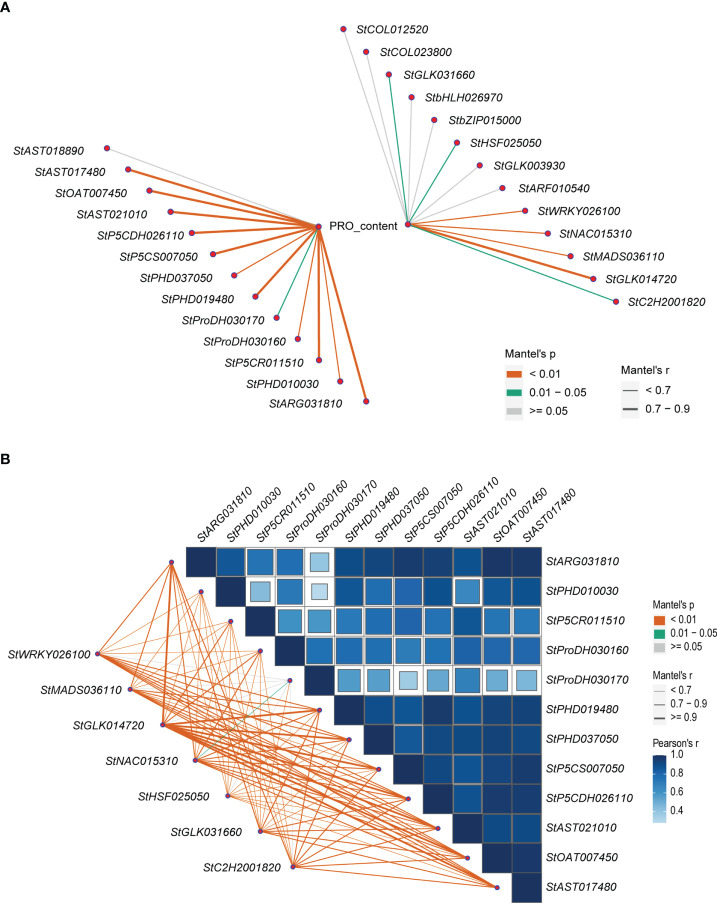
Correlation maps related to the proline contents, the expression levels of the structural DEGs and the TF-hub genes. **(A)** The correlations between the proline contents and the expression levels of the 13 structural DEGs in the proline metabolic pathway and the expression levels of the 13 TF-hub genes. **(B)** The correlations among the expression levels of the 12 structural DEGs in the proline metabolic pathway and between the expression levels of the 7 TF-hub genes and the 12 structural DEGs in the proline metabolic pathway. The lines correspond to the expression correlations between the 7 TF-hub genes and the 12 structural DEGs. The colors of the lines represent the Mantel’s *p*, and the thicknesses of the lines represent the percentage of the Mantel’s r. The intensity of the blue color and the size of the box indicate the degree of the expression correlation among the 12 structural DEGs.

Correlation analysis showed that the expression levels of 12 (92.3%) of the 13 structural DEGs in the proline metabolic pathway were significantly positively correlated with proline contents (Mantel’s r = 0.33~0.82, Mantel’s *p* < 0.05). Eight of them were Mantel’s r ≥ 0.7, including *StARG031810*, *StP5CR011510*, *StPHD019480*, *StP5CS007050*, *StP5CDH026110*, *StAST021010*, *StOAT007450*, and *StAST017480* (**Figure 5A**). Meanwhile, the correlations between the proline contents and the expression levels of the 13 TF-hub genes obtained from WGCNA analysis were also analyzed (**Figure 5A**). The results showed that the expression levels of 7 of the 13 TF-hub genes were significantly positively correlated with the proline contents (Mantel’s r = 0.29~0.75, Mantel’s *p* < 0.05). These genes were comprised of GLK family encoding genes *StGLK031660* and *StGLK014720*, HSF family encoding genes *StHSF025050*, NAC family encoding genes *StNAC015310*, WRKY family encoding genes *StWRKY026100*, C2H2 family encoding gene *StC2H2001820*, and MADS-MIKC family encoding gene *StMADS036110*. There was only one gene *StGLK014720* with Mantel’s r ≥ 0.7.

Then, the correlations between the expression levels of the 7 TF-hub genes significantly positively correlated with the proline contents, and the 12 structural DEGs in the proline metabolic pathway were further analyzed. The results showed that among the seven TF-hub genes, except for *StHSF025050*, the expression levels of each of the other six TF-hub genes were significantly positively correlated with that of 6~10 structural DEGs in the proline metabolic pathway (Mantel’s r ≥ 0.7, Mantel’s *p* < 0.01) (**Figure 5B**).

Interestingly, we found that the expression levels of each of the four TF-hub genes *StGLK014720*, *StNAC015310*, *StWRKY026100*, and *StC2H2001820* were highly significantly and positively correlated with those of eight of the same structural DEGs in the proline metabolic pathway (Mantel’s r ≥ 0.7, Mantel’s *p* < 0.01), and the highest correlations were found between the TF-hub gene *StGLK014720* and the eight of the same structural DEGs in the proline metabolic pathway (Mantel’s r > 0.9, **Supplementary Table 9**), indicating that the *StGLK014720* could be used as a key regulatory gene in the proline metabolic pathway under salt stress. Meanwhile, except for the structural DEG *StPHD037050*, the expression levels of the remaining 7 structural DEGs in the proline metabolic pathway were highly positively correlated with the proline contents (Mantel’s r ≥ 0.7, Mantel’s *p* < 0.01) (**Figure 5A**).

### The protein StGLK014720 could activate the promoters of both structural genes *StAST021010* and *StAST017480*

**Figure 6:**
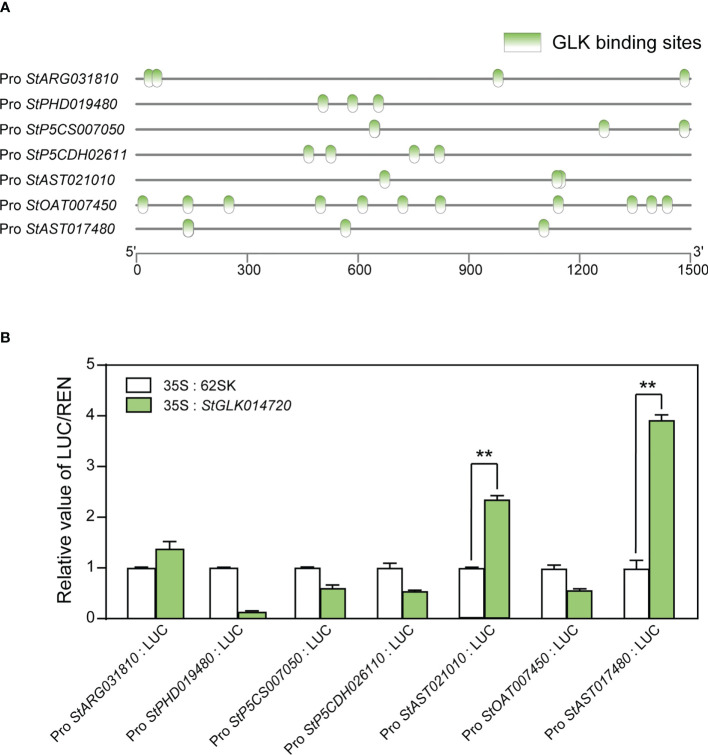
TFBs prediction and dual luciferase reporter assay between the protein StGLK014720 and the seven structural DEGs in the proline metabolic pathway. **(A)** Prediction of GLK binding sites in the promoter regions of the 7 structural DEGs in the proline metabolic pathway. **(B)** The ratios of LUC activity relative to REN activity represent the relative activities of the proline structural gene promoters. The LUC/REN ratios of the control were set to 1, to which the ratios of other groups were normalized. Data presented as means ± standard error from three biological replicates were tested by the Student *t*-test (** *P* < 0.01).

To predict the existence of TF binding sites (TFBs) in the promoter regions of the seven structural DEGs in the proline metabolic pathway, PlantPAN 2.0 database was used to analyze TFBs in gene promoter regions (1.5 Kb upstream of TSS). The results showed that the promoter regions of each of the seven structural DEGs presented multiple TFBs known to be recognized by all the four TF families. The largest number of GLK TFBs was predicted in the promoter region of the structural gene *StOAT007450*, with a total of 11 sites (**Figure 6A**). For the other three TF families NAC, C2H2, and WRKY, the numbers of TFBs to all the seven structural genes were 2~18, 3~7, and 4~30, respectively (**Supplementary Figure 8**).

To determine whether the key regulatory protein StGLK014720 can activate the promoters of the seven structural DEGs, a dual luciferase (LUC) assay was performed. *StGLK014720* driven by the CaMV35S promoter was used as an effector, while the promoters of the seven structural genes fused with LUC were used as the reporters. When the tobacco leaves co-transformed with the effector and the reporters, the LUC/REN ratios of the *StAST021010* and *StAST017480* were 2.2 and 3.9, respectively, and were drastically elevated when compared to the controls (*P* < 0.01, **Figure 6B**). These results indicated that the regulatory protein StGLK014720 could activate the promoters of both structural genes *StAST021010* and *StAST017480*.

## Discussion

### Biosynthesis and degradation of proline under salt stress

**Figure 7:**
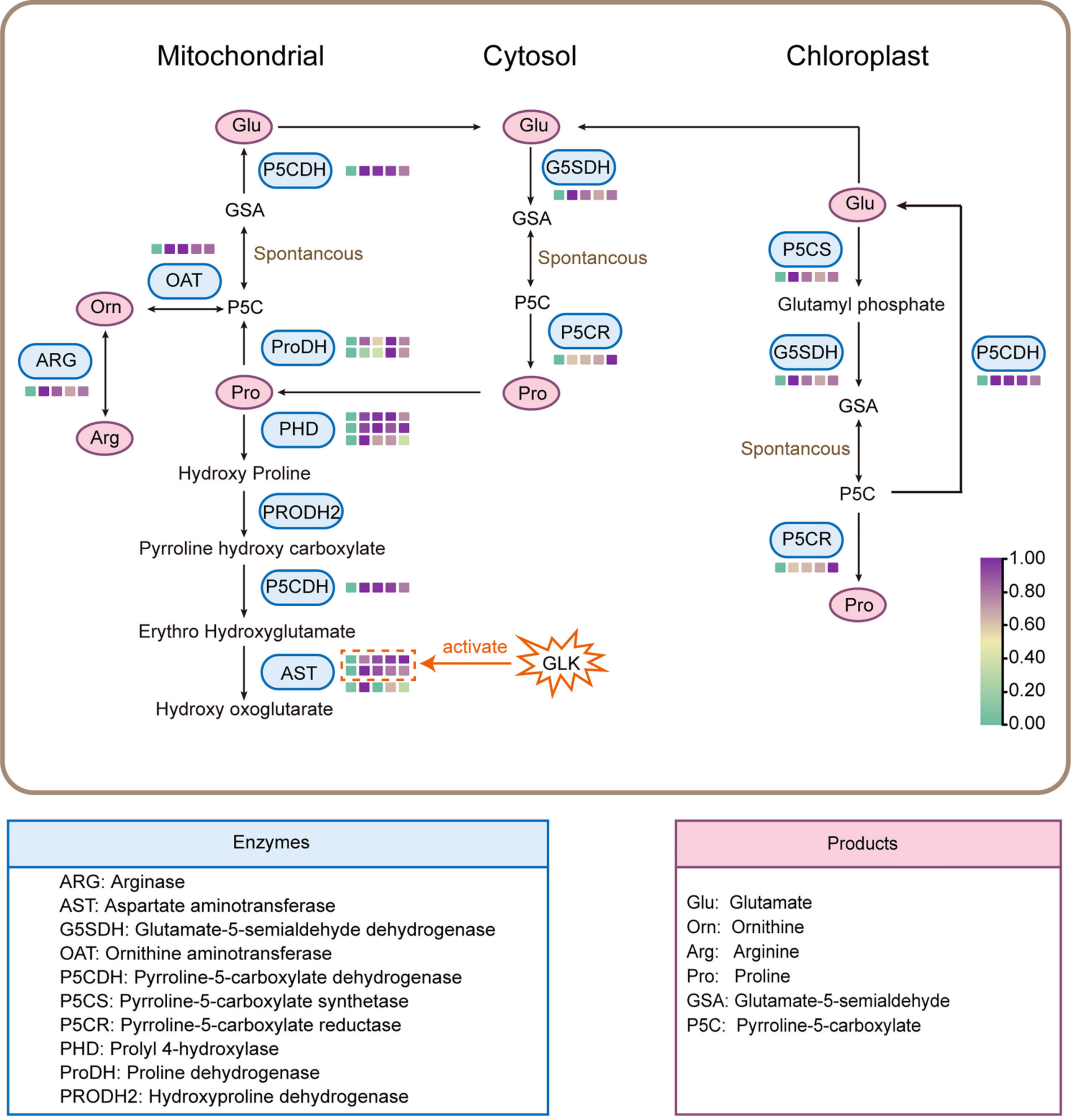
Proline biosynthesis and degradation pathway. The color of the box (□) represents the gene expression in different NaCl treatments.

Under salt stress, an increase in proline content was noted in many plants (Ghosh et al., 2022). A study in wheat supported that a high level of salt stress induced a sharp increase in the proline concentration (Hinai et al., 2022). The same type of response was noted for the rapid accumulation of free proline in salt-tolerant wild rice (Nguyen et al., 2021). In addition, the proline content was significantly increased throughout the experiment as the salt concentration increased in tomato seedlings (Shin et al., 2020). The results of our study indicated that the proline accumulation was also significantly increased in the aboveground parts of DM test-tube plantlets. Proline accumulation is achieved mainly by promoting proline biosynthesis in the cytosol and by inhibiting proline degradation in mitochondria (Lebreton et al., 2020). Proline biosynthesis can take place in different subcellular compartments such as the cytosol and chloroplast, and it is accomplished by different metabolic pathways such as the glutamate or ornithine pathway, depending on environmental conditions (Szabados and Savouré, 2010). In this study, the 13 structural DEGs identified in the proline metabolic pathway will contribute to further research on the molecular mechanism of proline accumulation in potato under salt stress (**Figure 7**).

Proline biosynthesis in most plant species occurs mainly from glutamate by the sequential steps of enzymes P5CS and P5CR (Furlan et al., 2020). In this study, it was found that the expression levels of genes *P5CS* and *P5CR* were significantly up-regulated in potato plantlets under salt stress, and the expression levels of the two genes were highly positively correlated with the proline contents, which was consistent with the results reported by Székely et al. (2008) in Arabidopsis, suggesting that genes *P5CS* and *P5CR* may promote the conversion of glutamate to proline. Hmida-Sayari et al. (2005) overexpressed the *P5CS* gene of *Arabidopsis Thaliana* in potato, and found that the proline accumulation and salt tolerance of transgenic potato lines were significantly enhanced. In addition, the ornithine pathway can also synthesize proline, which was initially transaminated by ornithine-delta-aminotransferase (OAT) producing GSA and P5C, and then converted to proline (Iqbal et al., 2014). We found that the expression levels of the *ornithine aminotransferase* (*OAT*) gene in the ornithine pathway and *arginase* (*ARG*) gene, which catalyzes the conversion of arginine to ornithine, were significantly up-regulated after salt treatment and highly positively correlated with the proline contents. Therefore, these results indicated that salt stress not only promoted the glutamate pathway, but also activated the ornithine pathway to achieve proline accumulation and thereby enhanced plant salt tolerance.

On the contrary, in the catabolic pathway, proline can be catalyzed by the sequential action of proline dehydrogenase (ProDH) and P5C dehydrogenase (P5CDH) to glutamate (Arabia et al., 2021). We identified several genes that promote proline decomposition in potato DEGs under salt stress, encoding ProDH, P5CDH, PHD, and AST, respectively. The house-keeping enzyme ProDH in proline degradation was inhibited in NaCl-treated wheat seedlings (Liu L. et al., 2020). However, in this study, all the structural DEGs in the proline catabolism pathway were up-regulated under salt stress. Therefore, it is speculated that the proline accumulation in the aboveground parts of DM test-tube plantlets might be achieved mainly by up-regulation of the proline biosynthesis pathway rather than by inhibition of the proline degradation pathway. In fact, activation of the proline catabolism was necessary for maintaining the growth of crop plants exposed to lengthy periods of stress (Kavi Kishor and Sreenivasulu, 2014). Although the expression patterns and functions of the structural genes in the proline metabolic pathway have been widely reported, the regulatory patterns are still unclear.

### Potential transcription factors regulating proline metabolism

Transcription factors (TFs) play a key role in response to salt stress. The results of our study showed that TF-coding genes in the 3691 DEGs shared among all the salt treatments were mainly distributed in the AP2/ERF, WRKY, MYB, and NAC families, which were consistent with the previous studies in rice and bermudagrass (Zhu et al., 2019; Shao et al., 2021). The four TF-hub genes obtained by WGCNA analysis were highly positively correlated with the expression levels of several structural DEGs in the proline metabolic pathway, and several TFBs corresponding to TFs were predicted at the promoter regions of the structural DEGs. Therefore, we speculated that the four TF-hub genes, including *StNAC015310*, *StWRKY026100*, *StC2H2001820*, and *StGLK014720*, might promote proline accumulation under salt stress by increasing the expression levels of the proline metabolic pathway genes.

NACs [no apical meristem (NAM), *Arabidopsis thaliana* transcription activation factor (ATAF1,2), and cup-shaped cotyledon (CUC2)] proteins belong to one of the largest plant-specific TF families and play important roles in plant development processes, response to biotic and abiotic cues, and hormone signaling (Singh et al., 2013). Wang et al. (2021) found that the NAC family gene *StNAC053* (*Soltu.DM.06G017300*) in potato was in response to salt stress, and the Arabidopsis transgenic lines over-expressing this gene had significantly enhanced tolerance to salt stress. Coincidentally, the novel gene *StNAC015310* in response to salt stress in this study also belongs to the NAC family and has the potential function of regulating the expression of genes in the proline metabolic pathway, and its salt tolerance needs to be further studied.

In plants, WRKY and C2H2 are important TF families that regulate a variety of biological processes and a wide range of biotic and abiotic stresses (Ahammed et al., 2020; Luo et al., 2022). The heterologous expression of the *Dioscorea composita* gene *DcWRKY3* strongly improved the salt tolerance of Arabidopsis transgenic lines, and the yeast one-hybrid (Y1H) assay verified the interaction between transcription factor DcWRKY3 and the promoter of proline synthesis gene *AtP5CS1* (Yu et al., 2022). Y1H and dual luciferase (LUC) assays showed that the C2H2 transcription factor RmZAT10, which promoted proline accumulation under salt stress, could interact with and activate the promoter of gene *RmP5CS*, and thereby elevate the expression level of gene *RmP5CS* (Luo et al., 2022). Therefore, the genes *StWRKY026100* and *StC2H2001820* discovered in this study might be novel genes in the regulation of the proline metabolic pathway, which had the potential function to regulate the structural gene *StP5CS007050* and yet to be confirmed in the near future.

The plant-specific GARP transcription factor family (composed of ARR-B and GLK type) contains genes with a variety of functions, including nutrient sensing, root and shoot development, floral transition, chloroplast development, circadian clock oscillation maintenance, hormone transport and signaling (Safi et al., 2017). However, GLK family proteins have not been reported in response to salt stress. Interestingly, our study found that the expression level of potato GLK family gene *StGLK014720* was highly positively correlated with that of the seven structural DEGs in the proline metabolic pathway (Mantel’s r > 0.9), and the protein StGLK014720 could activate the promoters of both AST encoding genes *StAST021010* and *StAST017480*, but not other structural DEGs, indicating that StGLK014720 may enhance the salt tolerance of potato plantlets by directly or indirectly regulating the AST protein expression at the transcriptional level (**Figure 7**). Increased AST activities under salt stress have previously been reported for different crop plants (Surabhi et al., 2008; Gao et al., 2013; Naliwajski and Sklodowska, 2018; Ullah et al., 2019). The up-regulated expression of both genes *StAST021010* and *StAST017480* may enable the potato plantlets exposed to the salt stress to maintain the efficient capacity of ammonia detoxification and proline homeostasis (Surabhi et al., 2008; Gao et al., 2013; Kavi Kishor and Sreenivasulu, 2014). However, further transgenic experiment is required to confirm the regulatory role of StGLK014720 under salt stress in potato plants.

## Data availability statement

The data presented in the study are deposited in the NCBI repository, accession number PRJNA882516 (https://www.ncbi.nlm.nih.gov/bioproject/PRJNA882516).

## Author contributions

XZ, ZL, QJ, and HH conceived and designed the experiments. QJ, XM, AC, and HZ performed the experiments. QJ, LW, and SZ analyzed the data. QJ, XZ, and ZL prepared the manuscript. XZ, ZL, and HH revised the manuscript. All authors contributed to the article and approved the submitted version.

## Funding

This research was supported by the National Natural Science Foundation of China (31771857), Anhui Provincial Natural Science Foundation (1808085MC65, 22232004), and Research Start-up Funds for High-level Personnels in Anhui Agricultural University (rc322101).

## Acknowledgments

The authors gratefully acknowledge Jun Sun of Anhui Agricultural University for providing the pGreen II 62-SK and the pGreenII 0800-LUC plasmids.

## Conflict of interest

The authors declare that the research was conducted in the absence of any commercial or financial relationships that could be construed as a potential conflict of interest.

## Publisher’s note

All claims expressed in this article are solely those of the authors and do not necessarily represent those of their affiliated organizations, or those of the publisher, the editors and the reviewers. Any product that may be evaluated in this article, or claim that may be made by its manufacturer, is not guaranteed or endorsed by the publisher.
