# Supplementary material for: Transcriptome analysis reveals the proline metabolic pathway and its potential regulation TF-hub genes in salt-stressed potato
Source: Front Plant Sci. 2022 Oct 17;13:1030138. doi: 10.3389/fpls.2022.1030138 (PMC9619106; doi:10.3389/fpls.2022.1030138)
Supplement: Supplementary file 1 [file DataSheet_1.zip › Supplementary Figures.docx]

# Supplementary Figures


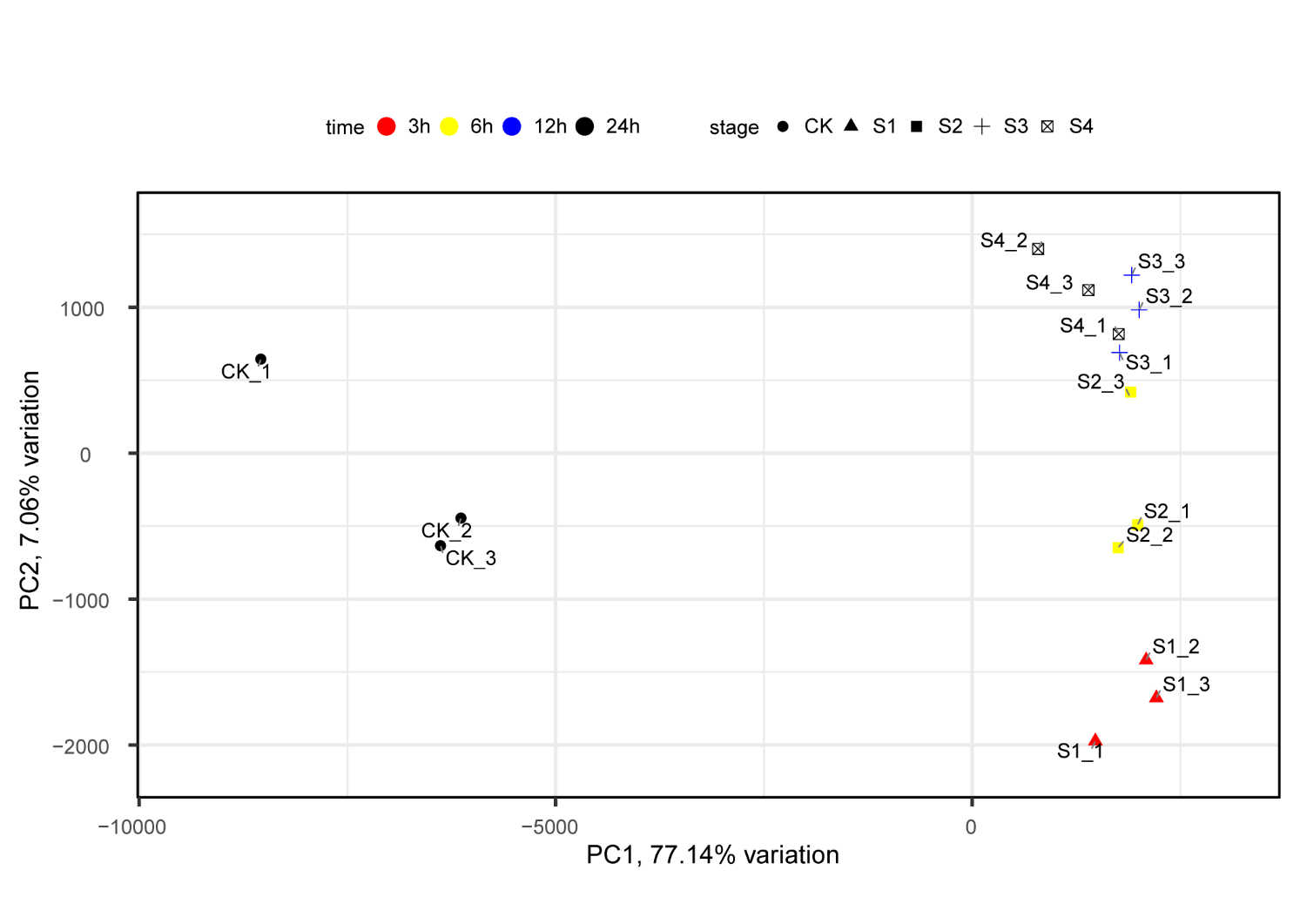


**Supplementary Figure 1. PCA analysis of the expression levels of the 15 samples in the DM plantlets.** Different colors represent different treatment times, and different shapes represent different treated samples.


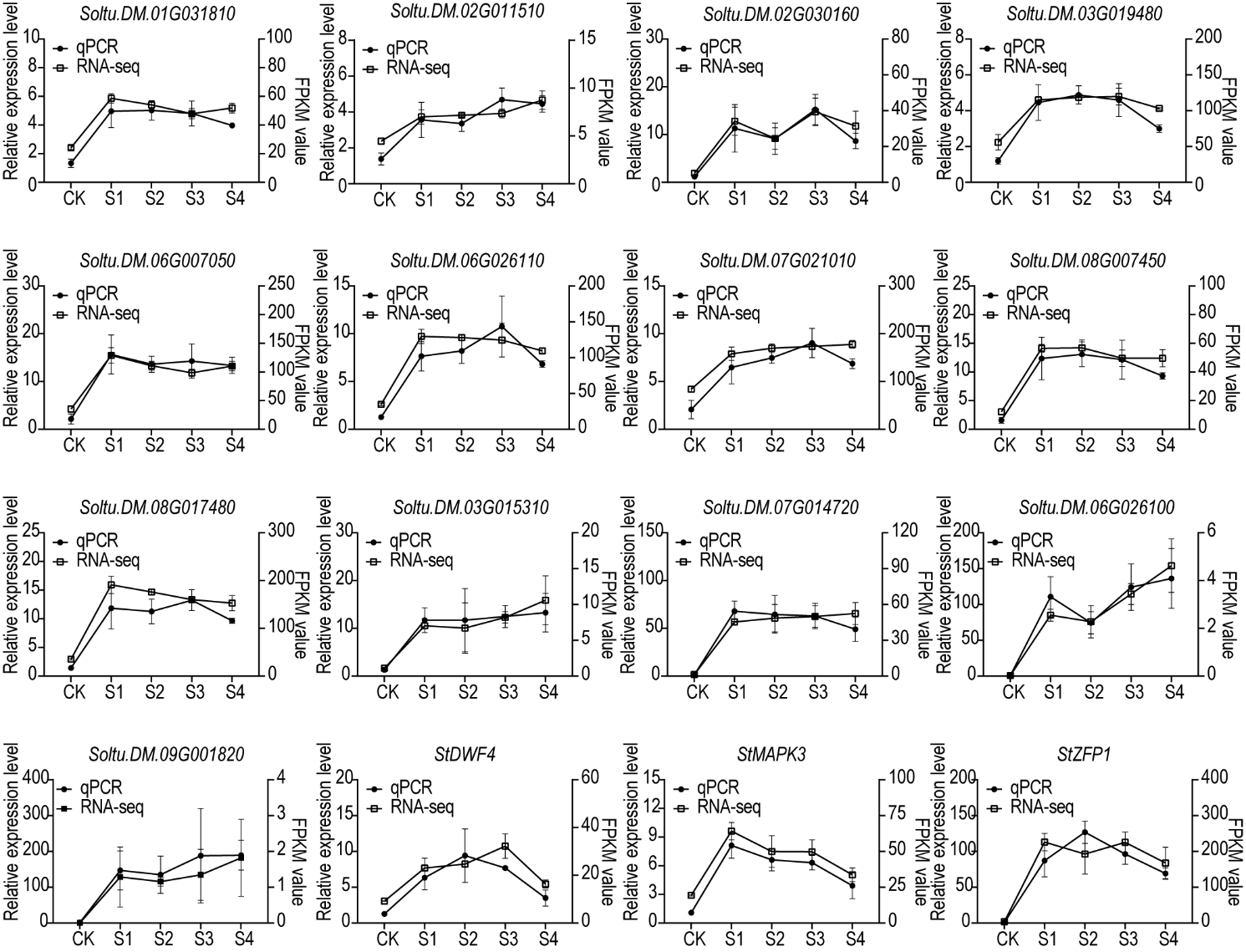


**Supplementary Figure 2. Comparison of qRT-PCR data and RNA-seq data of 16 genes.** The *StActin97* gene was used as the reference gene in qRT-PCR; the Y-axis on the left was the relative expression level of the gene for qRT-PCR, and the Y-axis on the right was the FPKM value for RNA-seq.


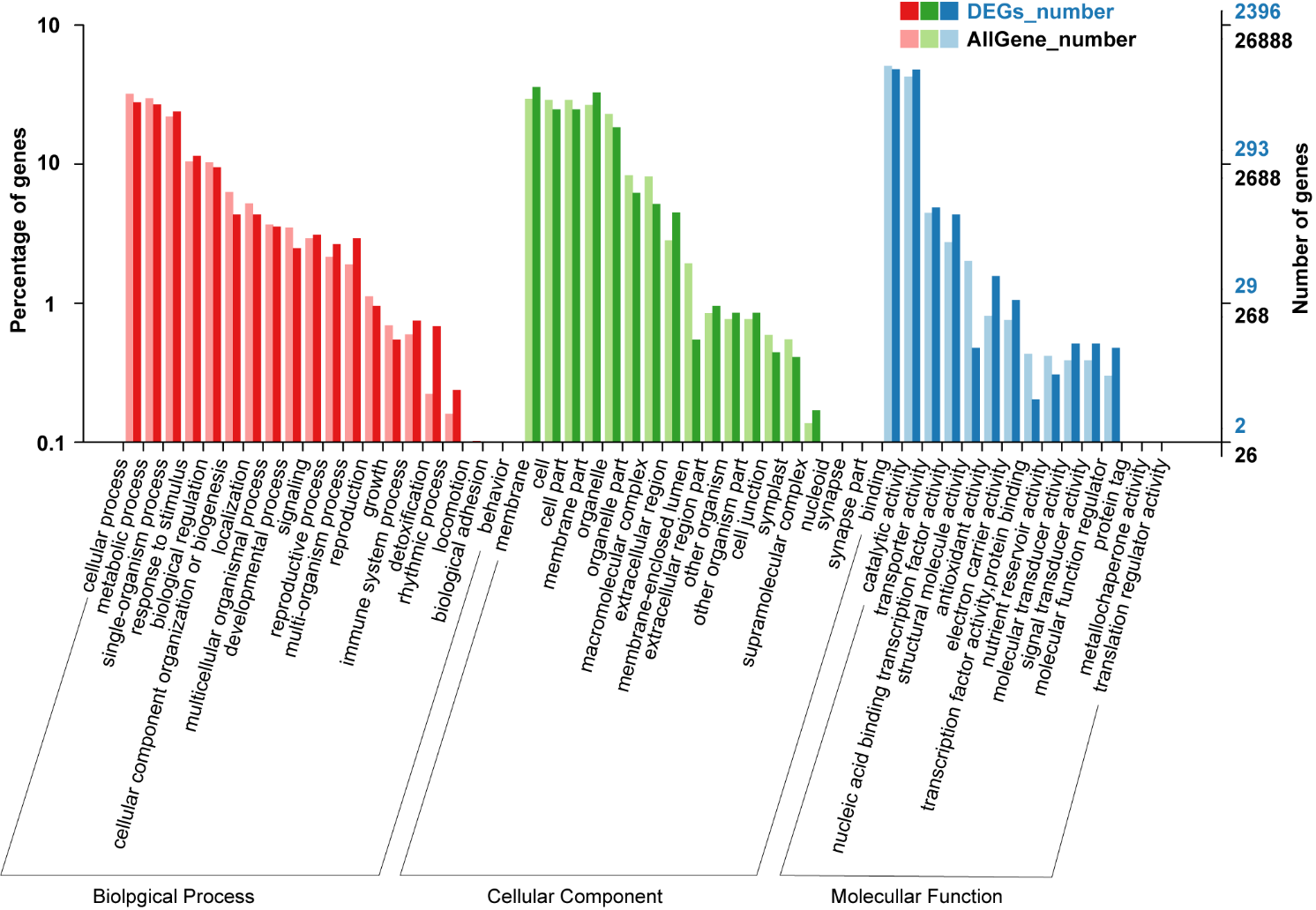


**Supplementary Figure 3. Histogram of GO classification.** The abscissa represents the GO classification; the left side of the ordinate represents the percentage of the number of genes; and the right side represents the number of genes. This figure shows the gene enrichment of each secondary function of GO under the background of DEGs and all genes, reflecting the status of each secondary function under the two backgrounds.


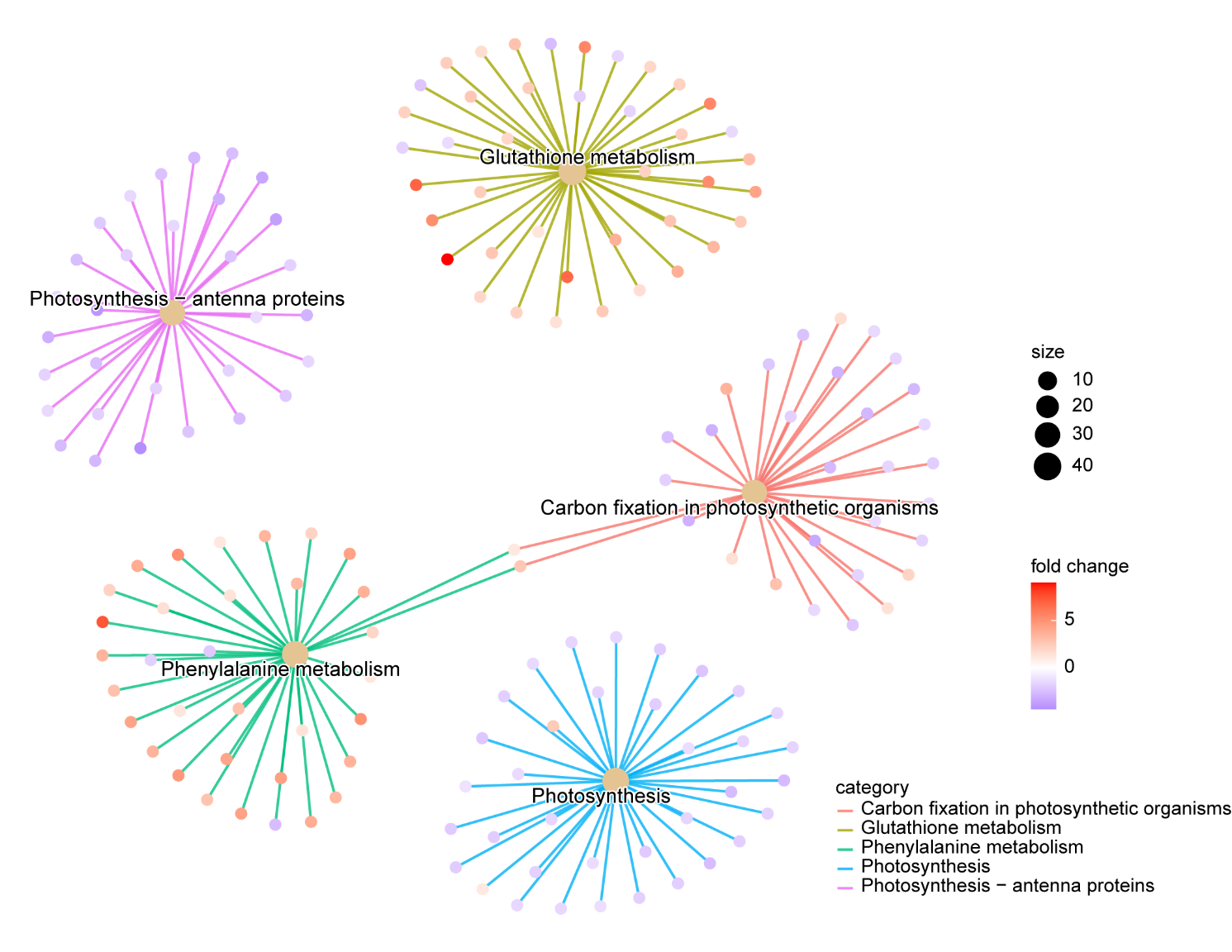


**Supplementary Figure 4. The DEGs are annotated to the main enriched Kyoto Encyclopedia of Genes and Genomes (KEGG) pathways in the DM plantlets.** The rich factor was the ratio of the DEG number to the total gene number in a certain pathway. The size of the pathway dots represents the gene number; the colour of the DEGs dots represent the fold change in gene expression.


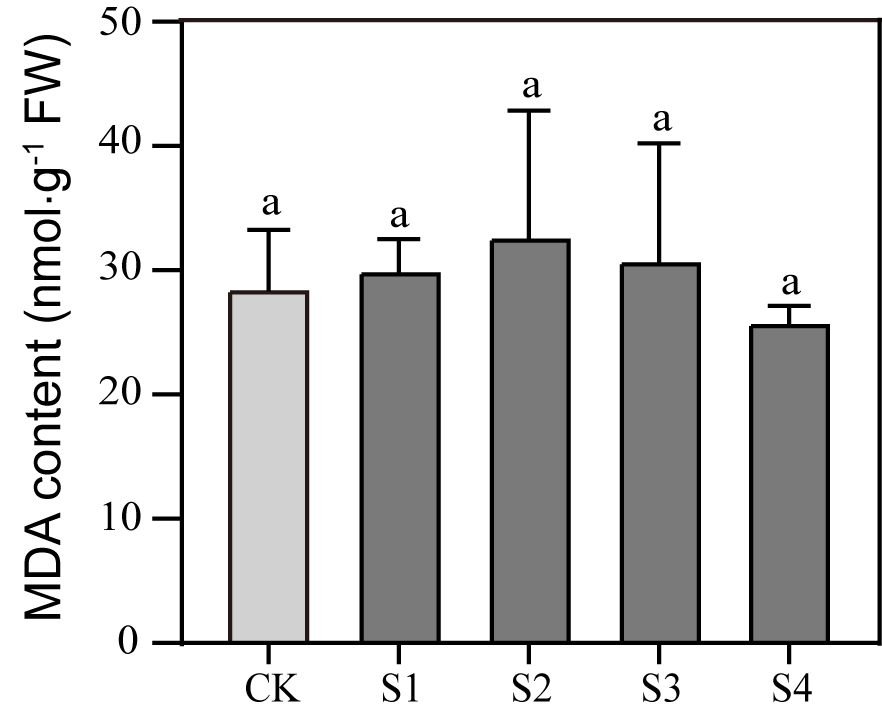


**Supplementary Figure 5. Malondialdehyde (MDA) contents in aboveground parts of DM test-tube plantlets under the 200-mM NaCl treatments.**


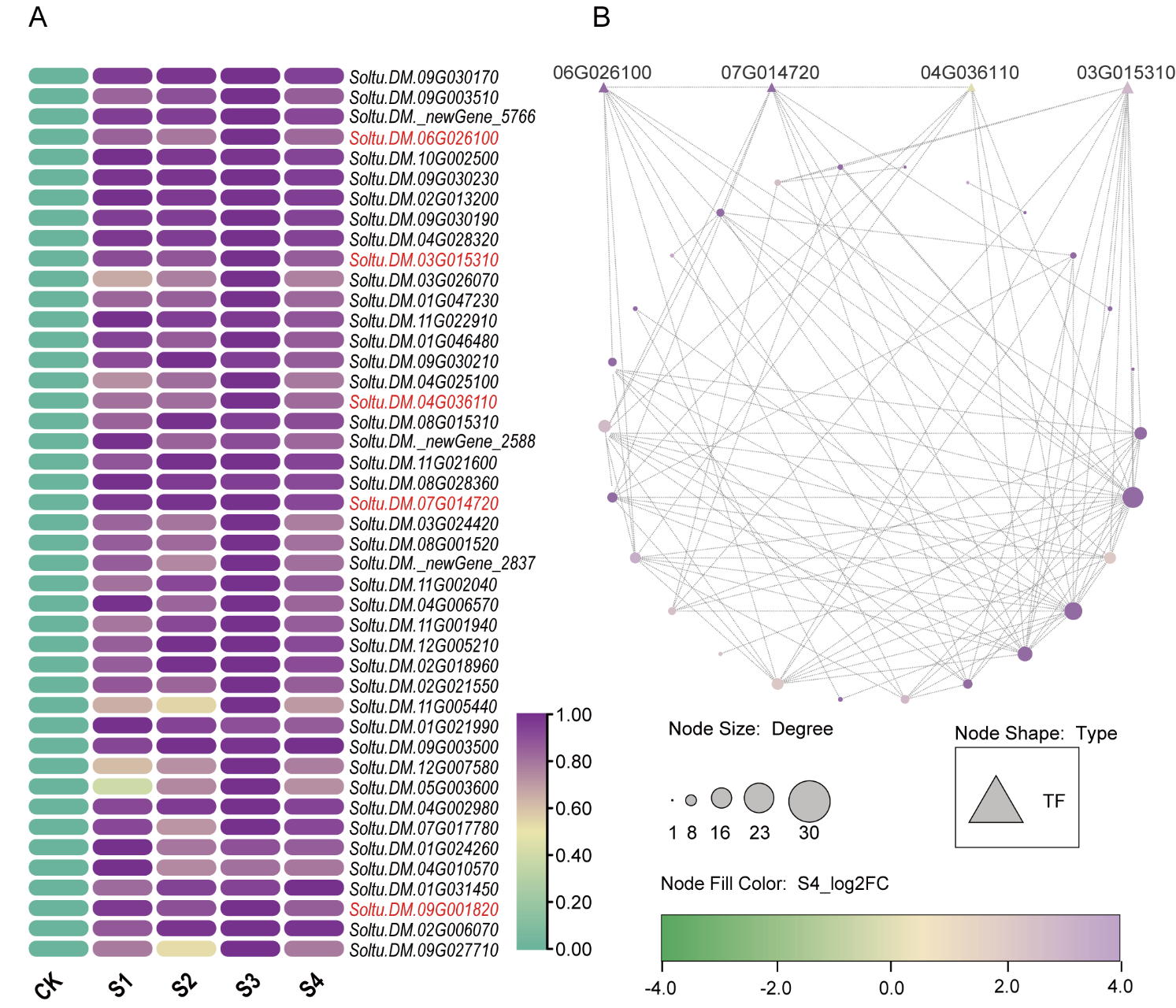


**Supplementary Figure 6. Characteristic gene expression patterns of the lightcyan module of WGCNA clustering.** **(A)** Gene expression heat map of the lightcyan module. The red gene ID represents TF-hub genes. **(B)** The network of co-expressed gene pairs in the lightcyan module. The shape and size represent the level of association in the network; the color represents the log_2_FC value of the S4 sample and the CK sample; the triangle represents the gene with the TF domain; the gene ID is simplified and the prefix “Soltu.DM.” is removed.


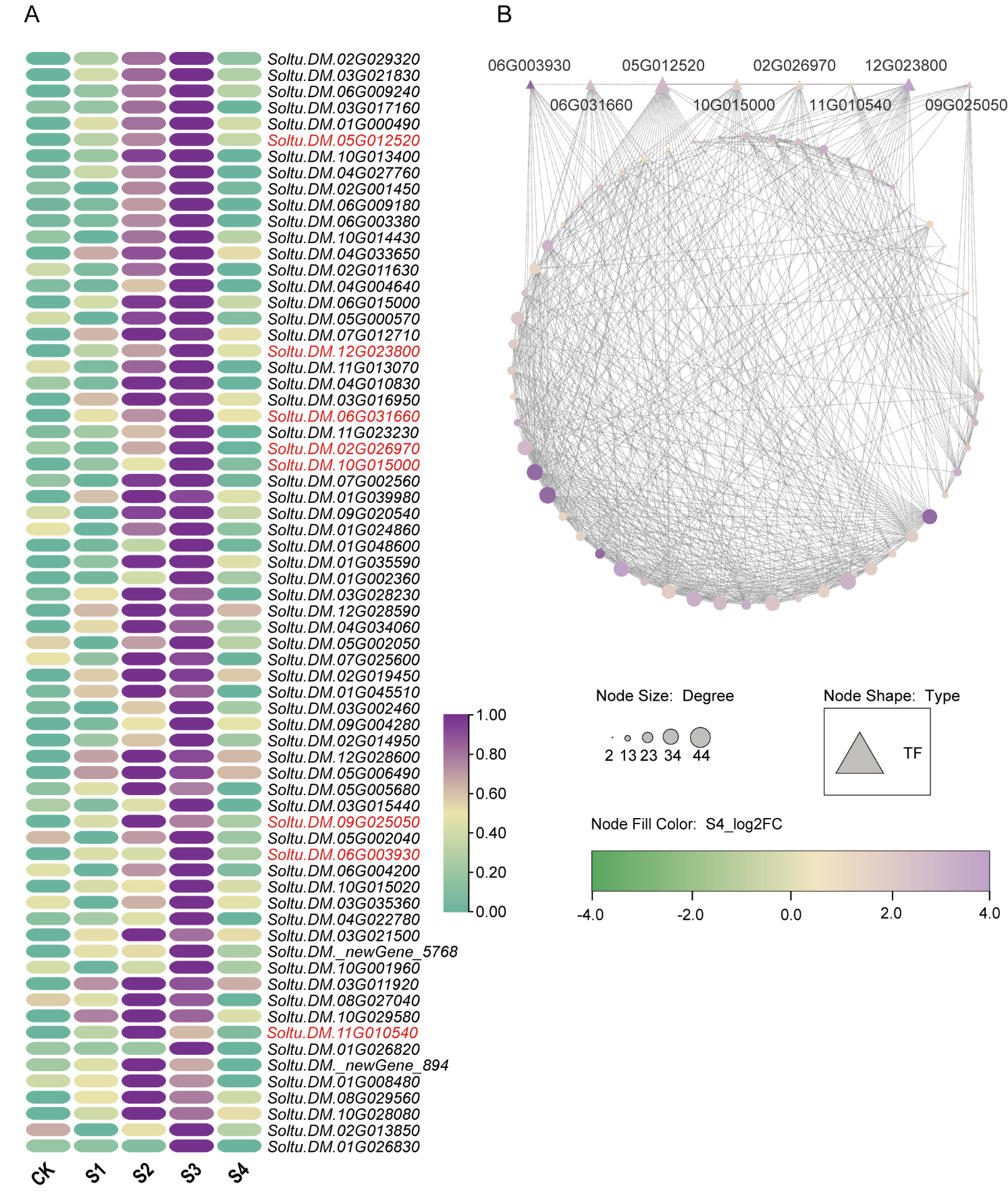


**Supplementary Figure 7. Characteristic gene expression patterns of the tan module of WGCNA clustering.** **(A)** Gene expression heat map of the tan module. The red gene ID represents TF-hub genes. **(B)** The network of co-expressed gene pairs in the tan module. The shape and size represent the level of association in the network; the color represents the log_2_FC value of the S4 sample and the CK sample; the triangle represents the gene with the TF domain; the gene ID is simplified and the prefix “Soltu.DM.” is removed.


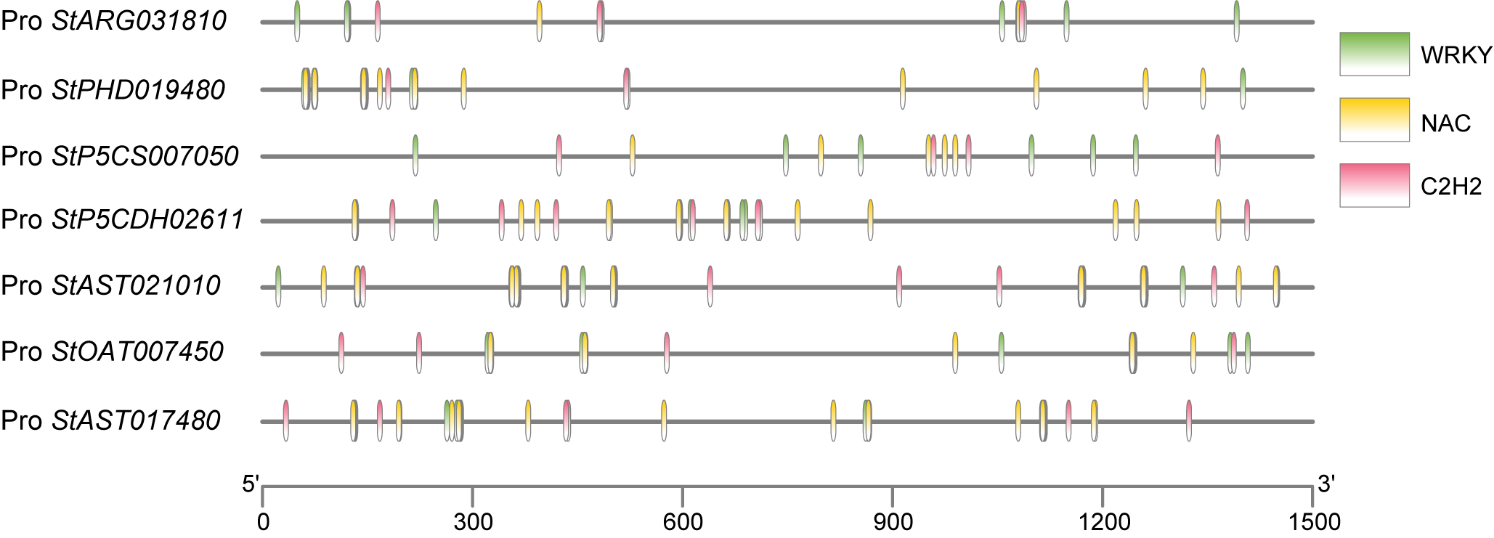


**Supplementary Figure 8.**  **Prediction of TF binding sites (TFBs)** **known to be recognized by the three TF families NAC, C2H2, and WRKY, in the promoter regions of the 7 structural DEGs in the proline metabolic pathway.**
